# Baseline values from the electrocardiograms of children and adolescents with ADHD

**DOI:** 10.1186/1753-2000-1-11

**Published:** 2007-09-28

**Authors:** Suyash Prasad, Amanda J Furr, Shuyu Zhang, Susan Ball, Albert J Allen

**Affiliations:** 1 Department of Neuroscience, Eli Lilly and Company Ltd., Basingstoke, Hampshire RG24 9 NL, UK; 2 Genzyme Therapeutics, Oxford OX4 2SU, UK; 3 Indiana University School of Medicine, Indianapolis, Indiana 46202, USA; 4 Lilly Research Laboratories, Eli Lilly and Company, Indianapolis, Indiana 46268, USA; 5 Department of Psychiatry, Indiana University School of Medicine, Indianapolis, Indiana 46202, USA

## Abstract

**Background:**

An important issue in pediatric pharmacology is the determination of whether medications affect cardiac rhythm parameters, in particular the QT interval, which is a surrogate marker for the risk of adverse cardiac events and sudden death. To evaluate changes while on medication, it is useful to have a comparison of age appropriate values while off medication. The present meta-analysis provides baseline ECG values (i.e., off medication) from approximately 6000 children and adolescents with attention-deficit/hyperactivity disorder (ADHD).

**Methods:**

Subjects were aged 6–18 years and participated in global trials within the atomoxetine registration program. Patients were administered a 12-lead ECG at study screening and cardiac rhythm parameters were recorded. Baseline QT intervals were corrected for heart rate using 3 different methods: Bazett's, Fridericia's, and a population data-derived formula.

**Results:**

ECG data were obtained from 5289 North American and 641 non-North American children and adolescents. Means and percentiles are presented for each ECG measure and QTc interval based on pubertal status as defined by age and sex. Prior treatment history with stimulants and racial origin (Caucasian) were each associated with significantly longer mean QTc values.

**Conclusion:**

Baseline ECG and QTc data from almost 6000 children and adolescents presenting with ADHD are provided to contribute to the knowledge base regarding mean values for pediatric cardiac parameters. Consistent with other studies of QT interval in children and adolescents, Bazett correction formula appears to overestimate the prevalence of prolonged QTc in the pediatric population.

## Background

The effect of medications on cardiac function, in particular the QT interval, has been an area of increasing focus in pediatric pharmacology. The QT interval is a measure of the period of depolarization and repolarization of the ventricles. Patients with a congenital or acquired condition of prolonged QT intervals, known as Long QT Syndrome, have a high incidence of cardiac events, syncope, and sudden death [1,2]. Prolonged QT intervals may be associated with fatal cardiac arrhythmias, such as torsades de pointes, and have therefore become a surrogate marker for a potential increased risk of cardiac sudden death [3].

Determination of whether the QT interval is prolonged can be made in reference to population norms. Because ECGs of pediatric patients differ from those of adults in a number of ways, specific normative data has to be established for this population. In one of the earliest studies with computerized ECG recordings, Davignon et al. [4] provided ECG values from over 2000 infants and children. Although this study is referenced frequently, the ECG parameter graphs and tables are not readily accessible as they were not published within the original article. Further, this dataset has been critiqued as being less applicable now due to changes in computerized technology and measurement standards as well as in patient populations (e.g., inclusion of non-whites, changes in mean values of height and weight) [5]. More recent population studies in children have been conducted in several countries. Fukushige et al., [6] analyzed ECG data from 4655 children in Japan at first and seventh grades; thus, measurements were taken only at 2 ages. Two smaller studies have examined children and adolescents across ages; one in the Netherlands (1912 subjects) [7] and one in Germany (373 subjects) [8].

Interpretation of whether the QT interval is prolonged is also dependent upon the method by which the interval measurement is corrected for heart rate (QTc). Correction is particularly important for pediatric patients because heart rate changes substantially during childhood development. At least 17 QT correction formulas have been suggested in the literature, but there is no universally accepted method [9]. Bazett's method is used most frequently but often results in an over-estimate of QT prolongation at higher heart rates. Fridericia's method is considered to be more appropriate at higher heart rates (as would be seen in pediatric populations) but may slightly underestimate cases of prolonged QT [10]. Others have suggested a correction based on values derived from repeated measurements of the population under study. In the data-derived method, the correction factor is the numeric value that results in a zero correlation between RR interval and corrected QT interval values. One limitation of the data-derived method is the feasibility of determination of multiple assessments with the same population; however, Wernicke et al., have provided a correction factor for the ADHD population based on analysis of repeated measures within 7 clinical trials [11].

The objective of the present meta-analysis is to provide descriptive values for the QTc interval based on approximately 6000 children and adolescents who presented with the neurodevelopmental condition attention-deficit/hyperactivity disorder (ADHD) and were physically healthy. ECG data were obtained as part of the global development and registration program for atomoxetine, which is a selective norepinephrine reuptake inhibitor used for the treatment of ADHD. A second goal of this study was to provide mean values based on different methods of correction for the QT interval. We used Bazett's method and the Fridericia method, which are the most common. We also included values using the data-derived formula for this specific population to illustrate how values from this method compare to the traditional formulas [11].

## Methods

### Subjects

Patients were children and adolescents aged 6 to 18 years who were recruited by referrals and advertisements. The studies included in the analyses were 20 clinical trials conducted in outpatient academic and private research centers in the United States, Canada, Puerto Rico, Europe, South Africa, Australia, and Israel. Subjects in this meta-analysis were being evaluated for participation in ADHD trials, but they were not excluded from the present study dataset if they did not meet their clinical trial's inclusion criteria. Subjects were diagnosed with ADHD as per the investigators clinical judgment, and then had to meet a minimum severity using the DSM-IV ADHD criteria. As the data obtained for this analysis was gathered at initial screen visits, subjects may or may not have met the criteria for ADHD. Of the total sample of 5930 entered subjects, 88% were eventually enrolled into their particular study. If a patient was currently on treatment for their ADHD, in order to enter the atomoxetine clinical trial program, they would need to have their baseline measurements taken while off all medication. Therefore, prior to screening and ECG recording, patients would have undergone a washout period equal to 5 half lives of their ongoing treatment.

Each study was conducted in accordance with the principles of the Declaration of Helsinki [12] and country specific ethical review guidelines. Each site's ethical review board independently reviewed and approved each study, and written informed consent to participate was obtained from the parent or guardian of each patient, as well as written assent from each patient.

### Procedures

Within the clinical trials, all patients underwent a comprehensive baseline evaluation of health status that included laboratory examination of blood and urine chemistries; clinical examination of vital signs, height, and weight; and a 12-lead electrocardiogram. The present analysis included ECG parameters collected from this baseline assessment prior to being assigned to treatment.

The ECG data were recorded at different investigator centers, but sent via direct transmission to a central ECG vendor, which used the Marquette 12SL ECG analysis program. The QT interval was measured in 3 leads on each 12-lead ECG: II, aVF, and V5. If any or all of the 3 leads were unmeasurable, alternate leads were used based on the pre-established sequence of: V3, V4, V6, I, III, AVL, V1, and V2. If sinus arrhythmia was present, the QT interval was the average of 5 different beats; in order to sample 5 different R-R intervals, QT was measured in II, aVF, V3, V4, and V5. If any or all of these 5 leads were unmeasurable, alternate leads were measured based upon the pre-established sequence of: V6, I, III, aVF, V1, and V2.

Within a trial, each ECG was read by the same pediatric cardiologist although cardiologists differed among the trials. For the cardiologist readings, the offset of a new QT interval was defined as the intersection of the line drawn along the downslopes of the T wave and the isoelectric line. U waves were ignored, but if a U wave or abnormal T wave obscured the offset of the T wave, then the offset of the QT interval was defined as the intersection of the tangent to the midpoint of the downslope of the T wave and the isoelectric baseline. Cardiologists made comments using the Marquette standard codes, which were then entered into the global safety database.

### Statistical methods

ECG parameters (heart rate; RR, PR, QRS, and QT intervals) and baseline characteristics were summarized for all entered patients. Subjects were classified as Caucasian or non-Caucasian, which included patients who identified themselves as being African, Hispanic, East Asian, or West Asian (Indian) origin. Between-group comparisons on continuous variables were assessed using an analysis of variance (ANOVA) with a term for the corresponding subgroup. All tests used a 2-sided significance level of .05.

As QT interval has an inverse relationship with heart rate, the measured QT intervals are usually corrected for heart rate in order to determine wether they are prolonged relative to baseline. Bazett and Fridercia are the most widely used methods of correction [11]. In addition a data-derived approach of the population under study, based on linear regression techniques may be most accurate [11]. We therefore used all 3 correction methods for the QT interval. In the Bazett method, the QT interval was divided by the square root of the R-R interval (defined as the length of the entire cardiac cycle). In the Fridericia formula, the QT interval was divided by the cube root of the R-R interval [10]. For the data-derived correction factor, we used the value that was determined in the Wernicke et al. study [11] by repeated ECG assessments of 2288 children and adolescents with ADHD. This data-derived factor of 0.38 falls between correction factors associated with the Bazett (0.50) and Fridericia (0.33) formulas.

Moss and Robinson [13], recommended that a QTc interval >460 ms be considered prolonged for women and children, as this value represents the top 1% of current, normal QTc distribution. Similarly, although the FDA acknowledges that no absolute agreement exists on the upper limit values for the QTc interval, in clinical trials, a QTc >500 ms has been identified as a concern [14]. Therefore, the proportions of patients with QTc >460 ms or QTc >500 ms were specifically identified and summarized for the entire sample. Mean QTc intervals using the different correction methods were also examined by pubertal grouping, which was defined by sex and age rather than by a medical assessment of whether the child had actually entered puberty. The prepubertal subjects consisted of females ≤ 8 years old and males ≤ 9 years old; the pubertal group was females between 8 and 13 years and males between 9 and 14 years; and the postpubertal group was females > 13 years and males > 14 years.

## Results

The entire sample comprised 5930 children and adolescents (Table 1); 5289 (89.2%) were from North America (US, Canada, Puerto Rico) and 641 (10.8%) were from non-North American countries. The non-North American group had a greater proportion of males and Caucasian patients than the North American group, although both groups demonstrated greater frequencies of males than females, as expected for the ADHD population. The mean age of the sample was approximately 10.7 years. Approximately 61.5% of the sample had a history of prior exposure to stimulant treatment.

**Table 1:** Demographics of Children and Adolescents Presenting with ADHD Across Clinical Trials by Region

	**North American **(*n *= 5289)	**Non-North American **(*n *= 641)
Country of origin: (n,%)		
United States	5046 (95.4)	
Canada	135 (2.6)	
Puerto Rico	108 (2.0)	
European		490 (76.4)
South Africa		71 (11.1)
Australia		47 (7.3)
Israel		33 (5.1)
Sex: n (%) Male	4013 (76)	572 (89)
Age: Mean years, SD	10.7 (2.6)	10.2 (2.3)
*Weight (kg): Mean, SD	40.5 (15.1)	37.4 (12.7)
Percentile (Mean, SD)	60.9 (29.2)	58.9 (28.9)
*Height (cm): Mean, SD	142.4 (16.8)	142.0 (14.5)
Percentile (Mean, SD)	51.4 (29.2)	57.8 (28.7)
Body Mass Index: Mean, SD	19.2 (4.0)	18.1 (3.4)
% Caucasian n (%)	4008 (76)	619 (97)

* Sample sizes for weight, height, and body mass were slightly lower

### Baseline mean values for ECG parameters by pubertal group

**Figure 1:**
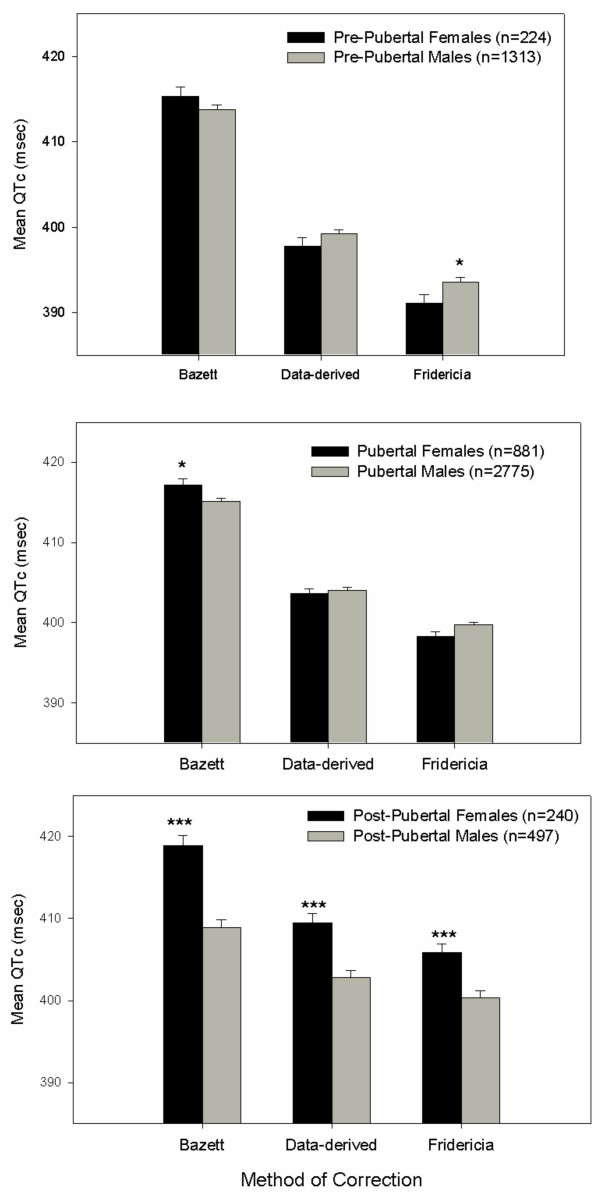
Mean QTc intervals based on 3 correction methods by age and sex for children and adolescents presenting with ADHD. Pre-pubertal: females ≤ 8 yrs (*n *= 224) males ≤ 9 yrs (*n *= 1313); Pubertal: females >8–13 yrs (*n *= 881), males >9–14 yrs (*n *= 2775); Post-pubertal: females >13 yrs (*n *= 240), males >14 yrs (*n *= 497). * *P *≤ .05, ***P ≤ 001.

In Tables 2, 3, 4, the mean values, standard deviations, and percentile scores for the ECG parameters are displayed by pubertal status. As developmentally expected, mean heart rate values decreased with age. Figure 1 shows the split diagrams of QTc by pubertal status and gender; the most consistent finding was in the post-pubertal age group, in which postpubertal males had significantly shorter QTc intervals than postpubertal females for each correction method (402.8 msec for postpubertal males vs 409.5 for postpubertal females, data-derived formula for QT correction, P ≤ .001, Figure 1).

**Table 2:** Mean and Percentiles of ECG Cardiac Measures Among Pre-pubertal Children

**ECG Variable**	**Mean (sd)**	**1^st ^%ile**	**5^th ^%ile**	**Median**	**95^th ^%ile**	**99^th ^%ile**
Heart Rate (bpm)	83.0 (12.1)	59.0	65.0	82.0	104	114.0
RR Interval (ms)	738.5 (107.8)	526.3	576.9	731.7	923.1	1040.0
PR Interval (ms)	132.8 (17.3)	100.0	108.0	130.0	162.0	180.0
QRS Interval (ms)	80.0 (9.0)	64.0	70.0	80.0	96.0	102.0
QT Interval (ms)	354.7 (24.3)	300.0	318.0	352.0	396.0	416.0
QTc Bazett (ms)	414.0 (19.6)	367.0	383.0	414.0	443.0	460.0
QTc Data-derived (ms)	399 (17.3)	357.4	372.0	398.7	424.1	439.6
QTc Fridericia (ms)	393.2 (17.1)	353.3	366.3	392.9	419.0	433.0

Note: Pre-pubertal group (N = 1537) defined as females whose age was ≤ 8 years (n = 224) and males whose age was ≤ 9 years (n = 1313)-

Abbreviations: RR, Duration of ventricular cardiac cycle (an indication of ventricular rate); PR, Time from the onset of atrial depolarization (P wave) to onset of ventricular depolarization (QRS complex); QTcb, Bazett's method for QT interval correction; QTcd, Data-derived corrected method for QT interval correction; QTcf, Fridericia's method for QT interval correction.

**Table 3:** Mean and Percentiles of ECG Cardiac Measures Among Pubertal Children

**ECG Variable**	**Mean (sd)**	**1^st ^%ile**	**5^th ^%ile**	**Median**	**95^th ^%ile**	**99^th ^%ile**
Heart Rate (bpm)	77.4 (11.8)	54.0	60.0	76.0	98.0	109.0
RR Interval	792.9 (121.1)	555.6	612.2	780.0	1000.0	1132.0
PR Interval (ms)	136.8 (18.5)	100.0	112.0	136.0	170.0	109.0
QRS Interval (ms)	83.0 (9.1)	68.0	70.0	80.0	96.0	106.0
QT Interval (ms)	368.8 (27.0.)	310.0	330.0	368.0	412.0	440.0
QTc Bazett (ms)	415.6 (21.3)	362.0	381.0	416.0	448.0	468.0
QTc Data-derived (ms)	403.9 (19.1)	357.0	373.3	404.0	433.1	450.1
QTc Fridericia (ms)	399.4 (19.0)	354.5	369.4	399.6	429.0	443.9

Note: Pubertal group (N = 3656) defined as females whose age was > 8 and ≤ 13 years (n = 881) and males whose age was > 9 and ≤ 14 years (n = 2775).

**Abbreviations: **RR, Duration of ventricular cardiac cycle (an indication of ventricular rate); PR, Time from the onset of atrial depolarization (P wave) to onset of ventricular depolarization (QRS complex); QTcb, Bazett's method for QT interval correction; QTcd, Data-derived corrected method for QT interval correction; QTf, Fridericia's method for QT interval correction.

**Table 4:** Mean and Percentiles of ECG Cardiac Measures Among Post-Pubertal Children

**ECG Variable**	**Mean (sd)**	**1^st ^%ile**	**5^th ^%ile**	**Median**	**95^th ^%ile**	**99^th ^%ile**
Heart Rate (bpm)	70.8 (11.4)	48.0	52.0	70.0	91.0	102.0
RR Interval	870.3 (144.3)	588.2	659.3	857.1	1153.8	1250.0
PR Interval (ms)	140.8 (18.9)	104.0	116.0	140.0	176.0	192.0
QRS Interval (ms)	87.8 (9.9)	70.0	72.0	88.0	104.0	112.0
QT Interval (ms)	382.9 (29.0)	328.0	340.0	380.0	432.0	470.0
QTc Bazett (ms)	412.2 (20.7)	350.0	376.0	413.0	444.0	457.0
QTc Data-derived (ms)	405.0 (18.5)	357.2	371.6	406.5	433.0	447.5
QTc Fridericia (ms)	402.1 (18.5)	354.5	371.1	402.6	430.9	446.5

Note: Post-Pubertal group (N = 737) defined as females whose age was >13 yrs (n = 240) and males whose age was > 14 yrs (n = 497)

Abbreviations: RR, Duration of ventricular cardiac cycle (an indication of ventricular rate); PR, Time from the onset of atrial depolarization (P wave) to onset of ventricular depolarization (QRS complex); QTcb, Bazett's method for QT interval correction; QTcd, Data-derived corrected method for QT interval correction; QTf, Fridericia's method for QT interval correction.

**Table 5:** Mean Values for ECG Parameters for Children and Adolescents with or without History of Stimulant Treatment

ECG Variable	Stimulant Naive (*n *= 2239) Mean (SD)	Stimulant Experienced (*n *= 3572) Mean (SD)	*P *value
Heart Rate (bpm)	77.5 (11.7)	78.3 (12.7)	*
RR Interval (ms)	791.3 (121.4)	786.6 (130.5)	
PR Interval (ms)	136.6 (18.7)	136.1 (18.3)	
QRS Interval (ms)	82.0 (9.0)	83.4 (9.6)	***
QT Interval (ms)	366.2 (26.2)	367.4 (29.0)	
QTc			
Bazett (ms)	413.1 (20.5)	415.9 (21.1)	***
Data-derived (ms)	401.4 (18.1)	403.7 (19.0)	***
Fridericia (ms)	396.8 (18.0)	399.0 (19.1)	***

* *P *≤ .05, ***P ≤ .001

**Table 6:** Mean Values for ECG Parameters for Caucasians and Non-Caucasians

ECG Variable	Caucasian (*n *= 4627) Mean (SD)	Non-Caucasian (*n *= 1303) Mean (SD)	*P *value
Heart Rate (bpm)	78.3 (12.5)	76.8 (11.7)	***
RR Interval (ms)	785.2 (127.8)	799.6 (123.4)	***
PR Interval (ms)	135.3 (18.2)	139.7 (18.8)	***
QRS Interval (ms)	83.5 (9.4)	80.8 (9.0)	***
QT Interval (ms)	366.8 (28.2)	367.2 (27.0)	
QTc			
Bazett (ms)	415.5 (20.8)	412.2 (20.8)	***
Data-derived (ms)	403.3 (18.7)	400.9 (18.7)	***
Fridericia (ms)	398.5 (18.7)	396.6 (18.6)	***

***P ≤ .001

### Baseline ECG mean values for subgroup populations

In addition to pubertal status, we also explored differences in QTc values based on prior treatment history. Patients who had a history of stimulant exposure had a small, but significantly greater heart rate compared with patients who had not received stimulants (77.5 bpm in treatment-naïve patients vs 78.3 bpm in stimulant-experienced patients, *P *≤ .05). The mean QTc values of the stimulant-experienced were slightly higher across the 3 correction methods compared with the stimulant-naive (*P *≤ .001, all comparisons; Table 5).

Baseline values also differed significantly based on racial origin. Comparisons between Caucasians and non-Caucasians children and adolescent showed that Caucasian subjects had significantly higher mean values in most ECG parameter except PR and QT intervals (Table 6). For each of the 3 correction methods, Caucasians also had significantly higher mean QTc values than did non-Caucasian subjects (*P *≤ .001, all comparisons).

### Frequencies of prolonged QTc intervals

The frequency of prolonged QTc was determined using the Moss and Robinson criteria of > 460 ms and the regulatory criteria of > 500 ms. With the criteria of a QTc >460 ms, the prevalence of QTc prolongation was 1.53% (91/5930) for the Bazett formula, 0.30% (18/5930) for the data-derived formula, and 0.27% (16/5930) for Fridericia's formula. With the criteria of QTc interval > 500 ms, the prevalence of prolongation was 0.12% (7/5930) for the Bazett formula, 0.12% (7/5930) for the data-derived formula, and 0.10 (6/5930) for Fridericia's formula.

Seven subjects had a QTc interval greater than 500 msec based on any 1 of the 3 correction formulas. For these cases, clinical information that was available from the screening visit was reviewed. The 7 patients had each been previously asymptomatic with no recorded history of syncope; 1 had a history of a heart murmur as an infant that was undetected upon physical examination during the screening; and 1 had sinus bradycardia and right axis deviation in addition to prolonged QT. The 7 subjects (6 males, 1 female) were pubertal age or younger (6 to 13 years) and did not vary from their pediatric norms in height, weight, BMI, or blood pressure; however, heart rate varied considerably. Two subjects had an elevated baseline heart rate: 150 bpm (age 11.1 years) and 103 bpm (age 7.2 years) compared with their age norms. QTc intervals greater than 500 msec was an exclusion criterion for the atomoxetine clinical trials; thus, none of these patients entered the clinical studies and were referred back to their family pediatrician for further medical care and follow-up as appropriate.

## Discussion

Understanding the distribution of cardiac parameters in children and adolescents is essential for the implementation of pediatric pharmacology. Indeed, considerable interest in the cardiovascular risks associated with ADHD medications exists from a regulatory perspective because of the high prevalence of ADHD and the widespread use of sympathomimetic agents that may increase blood pressure, heart rate, and cardiac rhythm parameters [15]. To ascertain changes in ECG parameters while on ADHD medication, it is important to have an understanding of the ECG values while off medication across the age range. Nonetheless, the determination of whether the QT interval is prolonged by a medication can be equivocal. For example, one regulatory definition of prolongation is a within-patient increase of 30 msec following initiation of a medication. However, in a meta-analysis of atomoxetine trials, 8.6% of patients who were taking placebo had at least a 30 msec increase [11]. Therefore, sole reliance on within-patient change may result in a high number of false positives, which suggests an alternative strategy that combines population norms based on sex and age as well as within-patient changes.

Age, sex, and racial origin were each factors that impacted mean baseline ECG values. Males in the post-pubertal age category were found to have significantly shorter QTc intervals than postpubertal females (402.8 msec vs. 409.5 msec, data-derived formula). Other studies also have shown that onset of sex differences in corrected QT occurs with puberty, but the reason for this difference and its clinical relevance is unclear [9]. With regard to racial origin, Caucasian children demonstrated faster heart rate, with correspondingly shorter ECG mean values, as well as slightly longer QTc intervals than did children and adolescents from other origins. This finding reflects genetic heterogeneity that may exist in physiology across ethnic groups [16].

There are a number of strengths and limitations of the meta-analysis. The considerable size and heterogeneity of the sample provides information that is not captured by current normative values in healthy children. Further, it represents the first report of ECG parameters from such a large sample of children who have a particular clinical condition, and the subgroup of females (N = 1345) is larger than previously reported female sample sizes from other studies. An additional strength of the study was the inclusion of 3 correction methods to allow for comparison of changes across age and sex. Mean values using the data-derived method were similar to those values observed with the Fridericia method. The prevalence rate when using the Bazett method and Moss and Robinson criteria were substantially higher (approximately 5-fold), and thus this formula may overestimate the true prevalence of prolonged QTc. Therefore, clinically, when a population derived correction factor may not be available, the optimum correction method for children would be the Fridercia method [17].

This meta-analysis also has several limitations. First, children were not formally assessed for their pubertal status, and the definitions for pubertal grouping in this meta-analysis were based on age rather than clinical examination. Another limitation is that the ECGs were computerized and were read by different cardiologists across trials. Computer-based methods have been criticized as demonstrating less sensitivity in detecting prolonged QT intervals [18]. The present study may underestimate or overestimate baseline prevalence rates, but these ECG assessments were measured using computer technology that is more contemporary than the methods used for currently referenced norms, such as those presented by Davignon et al. [4].

In evaluating this dataset, several characteristics regarding the subject population need to be considered. First, children under 6 years of age were excluded, which precludes this dataset as a population norm for all children. Similarly, the population assessed in this study was predominantly male. Although this could be considered a weakness of the analysis, it is reflective of the higher male incidence within the ADHD population. In addition, given the prevalence of ADHD (3 to 5%) [19], it is one of the most common conditions involved in pediatric pharmacology and thus of considerable public health importance [20]. A final consideration is that although these children and adolescents were overall physically healthy, the majority met criteria for ADHD. Psychopathology could influence cardiac parameters via changes in autonomic tone although ADHD has not been associated with increased stress responsivity [21].

As a representative sample of children and adolescents with ADHD, the values from this meta-analysis can be utilized in the context of clinical decision making. With increasing recognition that prolonging the QT interval may be one of a number of risk factors associated with serious adverse events, medications are being scrutinized for their effects on cardiac parameters. In recent years, 9 medications have been withdrawn or have received the cautionary "black box warning" by regulatory agencies due to concerns about cardiac effects [22]. For the population disease under study, ADHD, tricyclic antidepressants are an off-label treatment intervention that has been questioned for adverse cardiac events in children [23]. In the present sample, prior stimulant history was associated with small, but significantly greater mean QTc values, although the clinical relevance of this finding is undetermined. The baseline values in this paper serve as useful reference for ADHD specialists and may assist clinical decision making with regard to determination of rhythm abnormality.

In summary, the present study provides important baseline descriptions of the ECG and QT intervals from children and adolescents across a number of geographical regions. This considerable dataset can certainly be generalizable to a population of children with ADHD, however, it may not be entirely generalizable to a non-ADHD, healthy pediatric population. Given that ADHD is the most common neurodevelopmental disorder in childhood, these baseline ECG measures are of particular value, and perhaps should be the standards for comparisons with subsequent ADHD populations. In addition, we believe that this meta-analysis serves as a useful example of how large research databases can be mined creatively to provide clinically relevant and valuable information.

## Competing interests

Dr. Prasad was affiliated with Eli Lilly and Company Ltd., Basingstoke, Hampshire, UK during the course of this study [current affiliation is Genzyme Therapeutics, Oxford UK]. Drs. Ball, Allen, and Ms. Zhang are employees and/or shareholders of Eli Lilly and Company, Indianapolis, Indiana, USA. Ms. Furr was a medical student supported as a summer intern by Eli Lilly and Company, Indianapolis, Indiana, USA.
